# A highly conserved transcriptional repressor controls a large regulon involved in lipid degradation in *Mycobacterium smegmatis* and *Mycobacterium tuberculosis*

**DOI:** 10.1111/j.1365-2958.2007.05827.x

**Published:** 2007-08-01

**Authors:** Sharon L Kendall, Mike Withers, Catherine N Soffair, Nicole J Moreland, Sudagar Gurcha, Ben Sidders, Rosangela Frita, Annemieke ten Bokum, Gurdyal S Besra, J Shaun Lott, Neil G Stoker

**Affiliations:** 1 Department of Pathology and Infectious Diseases, The Royal Veterinary College Royal College Street, London NW1 0TU, UK.; 2 Laboratory of Structural Biology and Maurice Wilkins Centre for Molecular Biodiscovery, School of Biological Sciences, University of Auckland Auckland, New Zealand.; 3 School of Biosciences, University of Birmingham, Edgbaston Birmingham B15 2TT, UK.; 4 Department of Infectious and Tropical Diseases, London School of Hygiene and Tropical Medicine London WC1E 7HT, UK.

## Abstract

The *Mycobacterium tuberculosis* TetR-type regulator *Rv3574* has been implicated in pathogenesis as it is induced *in vivo*, and genome-wide essentiality studies show it is required for infection. As the gene is highly conserved in the mycobacteria, we deleted the *Rv3574* orthologue in *Mycobacterium smegmatis* (*MSMEG_6042*) and used real-time quantitative polymerase chain reaction and microarray analyses to show that it represses the transcription both of itself and of a large number of genes involved in lipid metabolism. We identified a conserved motif within its own promoter (TnnAACnnGTTnnA) and showed that it binds as a dimer to 29 bp probes containing the motif. We found 16 and 31 other instances of the motif in intergenic regions of *M. tuberculosis* and *M. smegmatis* respectively. Combining the results of the microarray studies with the motif analyses, we predict that *Rv3574* directly controls the expression of 83 genes in *M. smegmatis*, and 74 in *M. tuberculosis*. Many of these genes are known to be induced by growth on cholesterol in rhodococci, and palmitate in *M. tuberculosis*. We conclude that this regulator, designated elsewhere as *kstR*, controls the expression of genes used for utilizing diverse lipids as energy sources, possibly imported through the *mce4* system.

## Introduction

The success of *Mycobacterium tuberculosis* as a pathogen (Corbett *et al*., 2003) lies partly in its ability to adapt to varying conditions within the host. This adaptation depends on the co-ordination of gene expression via the regulation of transcription; in *M. tuberculosis* this is achieved by the collective action of the 190 transcriptional regulators that the genome encodes (Cole *et al*., 1998; Camus *et al*., 2002). The importance of these genes in pathogenesis is illustrated by the observations that in many cases, inactivation of genes encoding sigma factors (Chen *et al*., 2000; Ando *et al*., 2003; Sun *et al*., 2004; Calamita *et al*., 2005) or two-component regulatory systems (Perez *et al*., 2001; Zahrt and Deretic, 2001; Parish *et al*., 2003; Malhotra *et al*., 2004; Rickman *et al*., 2004; Martin *et al*., 2006; Walters *et al*., 2006) causes severe attenuation *in vivo*. However, the identities of the genes controlled by the majority of the transcription factors, and the functional roles of these genes *in vivo*, remain largely unknown.

The application of microarray technology to the study of bacterial gene expression during infection has allowed genome-wide analyses of genes important in pathogenesis. We previously reported a meta-analysis (Kendall *et al*., 2004) of data from studies in *M. tuberculosis*, and showed that there was (surprisingly) very little correlation between the lists of genes that were induced during infection (Schnappinger *et al*., 2003; Talaat *et al*., 2004) and those that were essential for infection (Sassetti and Rubin, 2003; Rengarajan *et al*., 2005). Indeed, only one gene was reported to be upregulated during macrophage infection, upregulated at the onset of acquired immunity in mice, and essential for infection in mice: *Rv3574*.

Rv3574 is a member of the TetR family of transcriptional regulators. These proteins are often repressors and are widely distributed among bacteria, regulating a number of diverse processes (Ramos *et al*., 2005). The prototype for this group is TetR from the Tn*10* transposon of *Escherichia coli*, which regulates the expression of a tetracycline efflux pump in Gram-negative bacteria (Orth *et al*., 2000). Other members of the TetR family include *Staphylococcus aureus* QacR, which regulates the expression of a multidrug transporter (Schumacher *et al*., 2001), and *M. tuberculosis* EthR, which regulates the expression of *ethA, a* monooxygenase that catalyses the activation of ethionamide, an antibiotic used in tuberculosis treatment (Baulard *et al*., 2000; Dover *et al*., 2004).

In this work we have examined the function of *Rv3574* in order to clarify the importance implied by our meta-analysis (Kendall *et al*., 2004). Our bioinformatic analyses indicate that *Rv3574* is highly conserved within the mycobacteria, and accordingly we have studied the function of orthologues in both *M. tuberculosis* and the fast-growing non-pathogen *M. smegmatis*. We inactivated the *Rv3574* orthologue in *M. smegmatis*, and used microarrays to identify a large number of genes that are de-repressed in the mutant. We identified a conserved regulatory motif present in the upstream regions of the genes in the regulon and also describe the same motif in *M. tuberculosis*. We show that recombinant *M. tuberculosis* Rv3574 binds as a dimer to short synthetic pieces of DNA containing this motif, and describe the likely regulons for *Rv3574* both in *M. tuberculosis* and in *M. smegmatis.* The functional relevance of the regulon in pathogenesis is discussed.

## Results

### *Rv3574* is a member of the TetR family of transcriptional regulators and is highly conserved in the mycobacteria

**Fig. 1:**
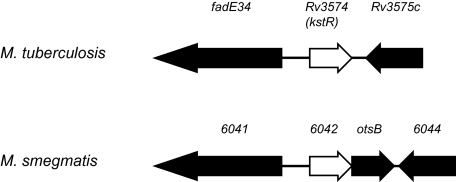
Conservation of the *Rv3574* region in the mycobacteria. *Rv3574* and its orthologue in *M. smegmatis* are shown in white, and other genes are shown in black. In all sequenced mycobacterial genomes and in *Nocardia farcinica,* a *fadE* gene encoding an acyl-CoA dehydrogenase was found adjacent to, but divergently transcribed from, *Rv3574* and its orthologues. The numbering for the *M. smegmatis* genes refers to the gene names (e.g. *6042* refers to *MSMEG_6042*).

Orthologues of *Rv3574* were identified through a combination of sequence similarity and synteny (the conservation of adjacent genes). In all cases, *Rv3574* and its orthologues are transcribed divergently from orthologues of the *M. tuberculosis fadE34*, encoding an acyl-CoA dehydrogenase (Fig. 1). The *Rv3574* region is highly conserved within the mycobacteria and is also conserved in the closely related species *Nocardia farcinica* (all > 70% amino acid identity over the whole length of the protein, and > 90% amino acid identity over the DNA binding domain). No convincing orthologue was found in the corynebacteria, while in *Streptomyces coelicolor*, a possible orthologue was found (*SCO2319*, 32% amino acid identity over the whole length of the protein) but with no conservation of synteny. In *M. leprae, Rv3574* is present as a pseudogene.

While we were writing this manuscript, a paper was published in which the *Rhodococcus* sp. strain RHA1 *Rv3574* orthologue is referred to as *kstR* (Van der Geize *et al*., 2007). In order to aid clarity when we discuss orthologues from different species, we will use this name hereafter, and discuss the relevance of their work later.

### Deletion of *kstR*_Msm_*(MSMEG_6042)* causes a defect in growth *in vitro*

A 646 bp pair deletion, removing the entire N-terminal DNA binding domain, was made in *kstR*_Msm_ producing strain ΔkstR1. Axenic growth of ΔkstR1 was compared with the wild-type strain and showed that, although the mutant grew at a similar rate to the wild-type, a slight increase in the lag phase was repeatedly observed (data not shown). In order to confirm that the phenotype was not caused by a second-site mutation, the experiment was repeated with an independently derived mutant, with similar results.

### Deletion of *kstR*_Msm_ leads to upregulation of adjacent genes

**Fig. 2:**
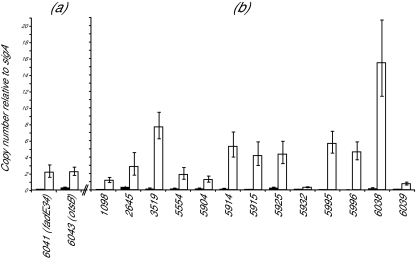
Changes in the expression levels of selected genes in the ΔkstR1 mutant compared with wild-type mc^2^155. The expression levels were measured in mid-log phase aerated cultures using RTq-PCR as described in *Experimental procedures*. The results are expressed relative to *sigA*, which was not significantly different in the mutant compared with the wild-type. Error bars represent ± 1 standard deviation. Filled bars, mc^2^155 wild-type; empty bars, ΔkstR1 mutant. A. Expression levels of genes adjacent to *kstR*. Both of the *fadE34* orthologue (*MSMEG_6041*) and *otsB* (*MSMEG_6043*) are significantly upregulated (de-repressed) in the mutant compared with the wild-type (unpaired Student's *t*-test; *P* ≤ 0.05). B. Expression levels of genes flanking 11 of the predicted KstR motifs in *M. smegmatis*. All genes tested were significantly de-repressed in the mutant compared with the wild-type strain, with levels of de-repression ranging from 6-fold (*MSMEG_5932*) to 155-fold (*MSMEG_6038*) (unpaired Student's *t*-test; *P* ≤ 0.05).

To examine whether *kstR*_Msm_ controls the expression of adjacent genes, the expression levels of the *fadE34* orthologue (*MSMEG_6041*) and *otsB* (Fig. 1) were measured in both wild-type and ΔkstR1 strains using real-time quantitative polymerase chain reaction (RTq-PCR). There is a 3 bp gap between the end of *kstR*_Msm_ and *otsB*, so these genes are likely to form an operon. The results (Fig. 2A) show that both *MSMEG_6041* and *otsB* are upregulated in the mutant strain (36-fold and 10-fold respectively). The experiment was repeated with the independently generated mutant, and confirmed the upregulation of *MSMEG_6041* and *otsB* in the mutant (data not shown). These observations suggests that *kstR*_Msm_ acts as a repressor of transcription of both *MSMEG_6041* and an operon consisting of itself and *otsB*.

### KstR_Mtb_ binds to a conserved motif within its own promoter region

**Fig. 3:**
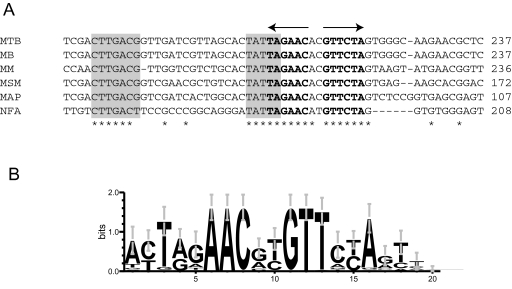
Identification of the KstR motif A. Alignment of the *kstR/fadE34* intergenic region in the mycobacteria and other closely related actinomycetes. Intergenic regions were aligned using ClustalW. Asterisks indicate residues conserved in all genomes. A conserved inverted palindromic repeat present in all species is shown in bold, with the direction of the palindrome indicated with arrows. Putative −35 and −10 regions are shaded in grey. MTB: *M. tuberculosis*; MB: *Mycobacterium bovis*; MM: *Mycobacterium marinum*; MSM: *M. smegmatis*; MAP: *Mycobacterium avium* subspecies *paratuberculosis*; NFA: *Nocardia farcinica*. B. Sequence logo of the KstR motif. Sequence logos (Crooks *et al*., 2004) show the relative frequency of each base at each position of the motif. The *y*-axis shows the information content, and error bars indicate an approximate, Bayesian 95% confidence interval.

TetR-like proteins normally bind to short palindromic DNA sequences (Grkovic *et al*., 1998; Orth *et al*., 2000; Ramos *et al*., 2005). Because protein binding constrains the evolution of these nucleotides, regulatory motifs may be identifiable through their conservation relative to neighbouring DNA sequences. We therefore aligned the intergenic region from *kstR*_Mtb_/*fadE34* (Fig. 1) from *M. tuberculosis* with the orthologous regions from other species, and found that there is an 18 bp region that is very highly conserved (Fig. 3A). Examination of the sequence showed that it contains a 14 bp palindrome [TAGAAC(N_2_)GTTCTA]. The other conserved nucleotides match known mycobacterial −10 and −35 regions (Gomez and Smith, 2000). The binding motif is upstream of, but partially overlapping, the −10 region, and this would efficiently block binding of the RNA polymerase.

**Fig. 4:**
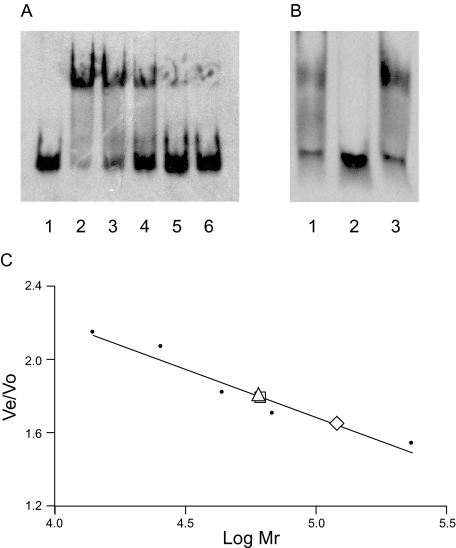
Purified KstR_Mtb_ binds to a 29 bp sequence containing the putative regulatory motif as a dimer. KstR_Mtb_ was expressed as a recombinant His_6_-tagged protein and purified using immobilized metal ion affinity chromatography, followed by SEC. A. EMSA of purified KstR_Mtb_ with a 29 bp fragment containing the highly conserved palindromic region (Table 2). A clear retardation of the 29 bp fragment is seen in the presence of His_6_-tagged KstR_Mtb._ The retardation decreases as the amount of protein used decreases. A total of 0.66 pmole of the labelled 29 fragment was used in the reactions with no protein (Lane 1), 17.2 pmole (Lane 2), 8.6 pmole (Lane 3), 4.8 pmole (Lane 4), 2.4 pmole (Lane 5) and 1.2 pmole (Lane 6) of protein. B. Specific and non-specific competition of protein–DNA interactions. The retardation seen with 4.8 pmole of protein (Lane 1) can be prevented by adding 100-fold excess of unlabelled probe (Lane 2), but not 150-fold excess of the non-specific inhibitor poly(dI-dC) (Lane 3). C. Molecular weight determination of the protein–DNA complex by SEC. The standard curve of v_*e*_/v_*o*_ versus the log Mr was derived from the peak elution volume (*v*_*e*_) of standard proteins. The void volume (*v*_*o*_) was determined using blue dextran 2000. The molecular masses of His_6_-tagged KstR_Mtb_, the 29 bp fragment, and the complex formed by them were calculated from the standard curve.

In order to determine whether KstR_Mtb_ binds directly to the motif we had identified, the protein was expressed as a His_6_-tagged form and used in electrophoretic mobility shift assays (EMSAs). His_6_-KstR_Mtb_ was purified by Ni^2+^-affinity chromatography, followed by size exclusion chromatography (SEC) to > 95% purity as judged by SDS-PAGE. The purified protein showed clear binding to the entire *kstR*_Mtb_/*fadE34* intergenic region (318 bp), but not to a random piece of DNA of the same size (data not shown). Additionally, the purified protein showed binding to a 29 bp DNA probe (Table 2: *Rv3573c/Rv3574* pair) containing the highly conserved palindromic region identified above. Figure 4A shows a clear retardation of the labelled 29 bp probe in the presence of increasing amounts of protein. This binding was lost with a 100-fold excess of unlabelled probe as a specific competitor, but a non-specific competitor did not abolish binding (Fig. 4B). These observations show that His_6_-KstR_Mtb_ binds directly and specifically within its own promoter region to a short region containing a highly conserved palindrome.

**Table 2:** Primers and oligonucleotides used in this study.

Primer	Sequence	Use
Δ*kstR*_Msm_ forward	ggAAGCTTaactgttcgcgcaccttc	Cloning *kstR*_Msm_ into p2NIL
Δ*kstR*_Msm_ reverse	ggGGATCCgggccatctactacgctcag	Cloning *kstR*_Msm_ into p2NIL
inv_*kstR*_Msm_ forward	gaggagcaacctaagatctagcgggtgagt	Making *kstR*_Msm_ deletion
inv_*kstR*_Msm_ reverse	ggAGATCTccgtgttggctgtgagtttccg	Making *kstR*_Msm_ deletion
pET_*kstR*_Mtb_ forward	cggCCATGGaagtggcggta	Cloning *kstR*_Mtb_ into pET30a for expression
pET_*kstR*_Mtb_ reverse	ggcgtaAAGCTTctaggcgctgtc	Cloning *kstR*_Mtb_ into pET30a for expression

	Primers used in RTq-PCR expression analysis	
	
Gene	Forward	Reverse
*MSMEG_1098*	gtcgctggagaccgtgtact	ttctcgtcgtggtccttctt
*MSMEG_2645*	cgcagtactgggtcatgaaa	gagtggatcggcgagtagaa
*MSMEG_3519*	gtacacccccgaacagctt	gtccatgccctcgtacttgt
*MSMEG_5555*	cactgttcgacaaccgtctg	tggtgtcatcttggtcttgg
*MSMEG_5904*	cgtcatgcgagttgaagttg	caccggatcggatttcac
*MSMEG_5914*	ctcggcactcgagaagagtt	aacacgcggtagatgtcctc
*MSMEG_5915*	ccaaggagtacggcctgat	tcacggatggtcttgaggat
*MSMEG_5925*	acacctacggcgagttcaag	cttccagatctcgacgtcct
MSMEG_*5932*	agacctcactcaacggcact	gaactgcaggtaacccttgc
*MSMEG_5995*	atcccttccgatttcgactt	gcctcgacacctccttgac
*MSMEG_5996*	gccgagacctactaccacctc	gttgacgttcaccttgtcca
*MSMEG_6038*	tcgatgagatcggcttcttc	cagttgtgcacaccgatgat
*MSMEG_6039*	tccacatcgaggtgttcaag	gtccagcagtacatcgagca
*MSMEG_6041*	gcttccggcacagtcgaatt	gggtgcgttgtccagctcg
*MSMEG_6043*	cgatttcgacggcacactcgc	ggcacgtccggagatcag
*MSMEG_2758 (sigA)*	ccaagggctacaagttctcg	cttgttgatcacctcgacca

	Oligonucleotides used in EMSAs	
	
Flanking genes	Forward	Reverse
*Rv0223c*	gcgaaacgagaacgtgttccattattagg	cctaataatggaacacgttctcgtttcgc
*Rv0551c_Rv0552*	tccaaattgcaacacgttctagtcttgcc	ggcaagactagaacgtgttgcaatttgga
*Rv0940c*	gtcaaattagaacacgttctaatctcgtt	aacgagattagaacgtgttctaatttgac
*Rv0953c/Rv0954*	gcgcaggtggaacacgttctaattcggtg	caccgaattagaacgtgttccacctgcgc
*Rv1894c/Rv1895*	atagaactgaaacgtgttctagtttagta	tactaaactagaacacgtttcagttctat
*Rv3503c/Rv3504*	cagagactagaacgtgttacaaccgggaa	ttcccggttgtaacacgttctagtctctg
*Rv3515c/Rv3532*	accaaactagaacgtgttacatttcttga	tcaagaaatgtaacacgttctagtttggt
*Rv3520c/Rv3521*	tgtcgagtagaacaggttctaacaacggt	accgttgttagaacctgttctactcgaca
*Rv3525c/Rv3526*	cgctaactagaacacgttacagttttctc	gagaaaactgtaacgtgttctagttagcg
*Rv3531c/Rv3532*	tcgacactagaacgtgttcctgttttgcg	cgcaaaacaggaacacgttcta
*Rv3545c/Rv3546*	atagtaatgaaacgtgttctagcctggcc	ggccaggctagaacacgtttcattactat
*Rv3570c/Rv3571*	gaaagactagaacacgttccgatttgtgt	acacaaatcggaacgtgttctagtctttc
*Rv3570c/Rv3571*	tcataactagaacatgttacagaaaaccc	gggttttctgtaacatgttctagttatga
*Rv3573c/Rv3574 (kstR)*	tgcccactagaacgtgttctaatagtgct	agcactattagaacacgttctagtgggca

### KstR_Mtb_ binds to the motif as a dimer

In order to study the binding stochiometry of His_6_-KstR_Mtb_ to the motif, the elution of the protein alone and in the presence of the 29 bp fragment was analysed by SEC and compared with a standard curve of *v*_*e*_* /v*_*o*_ versus log Mr (Fig. 4C). The molecular mass of His_6_-KstR_Mtb_ was determined to be 60.2 kDa, which is consistent with the protein forming a dimer in solution (the predicted monomeric molecular mass is 27.7 kDa). The apparent molecular mass of the 29 bp fragment alone was determined to be 58.9 kDa; note that this substantially exceeds its actual mass of 18.0 kDa due to the inflexible rod structure of DNA in comparison with the globular shape of standard proteins (Reuter *et al*., 1998). Only one species of KstR_Mtb_–DNA complex with an apparent molecular mass of 118.7 kDa was detected at protein:DNA ratios of 1:4, 1:1 and 4:1. This is consistent with a complex of dimeric His_6_-KstR_Mtb_ bound to one 29 bp fragment of DNA. Dimeric binding to palindromic DNA is characteristic of the TetR family of transcriptional regulators (Huffman and Brennan, 2002). Although we cannot exclude the possibility that an alternative DNA conformation is assumed in the protein–DNA complex, altering its apparent mass, structural analyses with TetR show that the DNA is generally straight (Huffman and Brennan, 2002), and we conclude that a dimeric state is the most likely.

### The motif is present in the upstream regions of other genes in both *M. tuberculosis* and *M. smegmatis*

The experiments described above show that His_6_-KstR_Mtb_ binds as a dimer to a 29 bp sequence within its own promoter that contains a highly conserved palindromic sequence overlapping a putative −10 region. This is consistent with it acting as a direct repressor of transcription, and indicates that the de-repression seen in the *M. smegmatis*ΔkstR1 strain is due to the loss of binding of KstR_Msm_ to its own promoter. In order to identify whether the palindromic sequence is present within the upstream regions of additional genes, we searched for the motif in both the *M. smegmatis* and *M. tuberculosis* genomes.

We first used the promoter regions of the *kstR* orthologues as a training set for the motif identification program MEME (Bailey and Elkan, 1994). This generated a motif profile that was used to search a database of intergenic regions from *M. tuberculosis* and *M. smegmatis* using the sister program MAST (Bailey and Gribskov, 1998). This predicted a total of 16 motif instances in *M. tuberculosis* and 31 in *M. smegmatis* (Table 3). Many of these are situated between divergently transcribed genes, while in the region between *Rv3570c* and *Rv3571* (and the orthologous genes *MSMEG_6038/MSMEG_6039*), there are two copies of the motif. Comparative genomics of *M. smegmatis* and *M. tuberculosis* showed that, of the genes associated with the motif, a number of them are orthologues and these are indicated in Table 3. The information was used to generate a sequence logo of the *kstR* motif (Fig. 3B), the core of which is the palindrome **T**nn**AAC**nn**GTT**nn**A**.

**Table 3:** Intergenic sequences in *M. smegmatis* and *M. tuberculosis* with significant matches to the palindromic KstR motif.

Motif sequence	*P*-value	Flanking genes	Experimental evidence
*Instances in* M. smegmatis			*RTq-PCR*
AT**TGGAAC**GT**GTTCTA**GTTC	1.5e-09	MSMEG_0305/MSMEG_0306	ND
AC**GAGAAC**CT**GTTCCA**GTTG	2.06E-07	MSMEG_0309^a^	ND
AC**TAGAAC**GT**GTTCCA**GAAA	2.19E-09	MSMEG_1098^b^	+ve
AC**GAGAAC**AC**GTTCTA**GTTG	4.93E-08	MSMEG_2645	+ve
AC**TGGAAC**GT**GTGGCA**ATAC	5.40E-07	MSMEG_2761/MSMEG_2763	ND
AC**TGAAAC**GT**GTTACA**GCCG	1.65E-07	MSMEG_2790	ND
AT**TAGAAC**CT**GTTCCA**ATTC	6.03E-09	MSMEG_3515/MSMEG_3516	ND
AT**TAGAAC**AC**GTTTCA**GTCT	5.41E-09	MSMEG_3519^f^	+ve
AT**CAGAAC**AC**GTTCCA**GAAA	6.96E-08	MSMEG_3522/MSMEG_3524	ND
TC**TGGAAC**AG**GTTCTA**GTTT	2.89E-08	MSMEG_3562	ND
AC**TGCAAC**AC**GTTTCA**GTTT	1.32E-07	MSMEG_3843^e^	ND
AT**TAGAAC**GT**GTTCTA**GTCG	1.24E-10	MSMEG_5202	ND
AC**TGGAAC**GT**GTTTCA**GTTA	4.14E-08	MSMEG_5228	ND
GT**TGCAAC**AC**GTTCTA**GCGA	2.57E-07	MSMEG_5232/MSMEG_5233	ND
AC**TGGAAC**AC**GTTCTA**CCAC	1.04E-07	MSMEG_5286	ND
AC**TGAAAC**AC**GTTCTA**ATTC	1.46E-08	MSMEG_5519/MSMEG_5520^d^	ND
TT**TAGAAC**AC**GTTCTA**GTGT	6.48E-10	MSMEG_5554/MSMEG_5555^c^	+ve
CT**TAGAAC**GT**GTTCCA**CCGC	3.90E-07	MSMEG_5579/MSMEG_5580	ND
AT**TAGAAC**GT**GTTCCA**GAAA	2.81E-09	MSMEG_5583/MSMEG_5584	ND
TC**TGAAAC**GT**GTTCTA**ACGG	1.04E-07	MSMEG_5902	ND
AC**TAGAAC**GT**GTTACA**ACCG	8.57E-09	MSMEG_5904^g^MSMEG^−1^_5906	+ve
AC**TAGAAC**GT**GTTACA**TTTC	3.45E-08	MSMEG_5914^h^/MSMEG_5915^i^	+ve
TC**TAGAAC**GC**GTTCTA**GACA	1.99E-08	MSMEG_5919	ND
CT**TAGAAC**CT**GTTCTA**CTCG	5.40E-07	MSMEG_5920^j^/MSMEG_5921^k^	ND
AC**TGTAAC**GT**GTTCTA**GTTA	9.47E-09	MSMEG_5925^l^	+ve
AC**TAGAAC**GC**GTTCTA**ATTC	2.89E-10	MSMEG_5932^m^MSMEG^−1^_5933	+ve
AC**TGAAAC**GT**GTTCTA**GCCT	2.61E-08	MSMEG_5995^n^/MSMEG_5996°	+ve
AT**TAGAAC**AC**GTTACG**ATTT	9.65e-08	MSMEG_6038^p^/MSMEG_6039^q^	+ve
TT**TGTAAC**CT**GTTCTA**GTTC	2.76E-07	MSMEG_6038^p^/MSMEG_6039^q^	+ve
AC**TAGAAC**GT**GTTCTA**ATAG	2.35E-11	MSMEG_6041^r^/MSMEG_6042^s^	ND
AC**TAGAAC**AC**GTTCTA**GTGA	7.20E-11	MSMEG_6475	ND
*Instances in* M. tuberculosis			*EMSA*
AC**GAGAAC**GT**GTTCC****A**TTAT	2.22E-07	Rv0223c^a^	–ve
AC**TAGAAC**GT**GTTGC****A**ATTT	6.03E-09	Rv0551c^b^/Rv0552	+ve
GT**TAGAAC**AC**GTTACA**GTTT	7.54E-08	Rv0687	ND
AT**TAGAAC**GT**GTTCTA**ATTT	7.20E-11	Rv0940c^c^	+ve
AT**TAGAAC**GT**GTTCCA**CCTG	2.17E-08	Rv0953c^d^/Rv0954	+ve
AC**TGAAAC**GT**GTTGC****A**GTTC	1.32E-07	Rv1628c^e^/Rv1629	ND
AC**TGAAAC**GT**GTTCTA**GTTT	6.83E-09	Rv1894c^f^/Rv 1895	+ve
AC**TAGAAC**GT**GTTACA**ACCG	8.57E-09	Rv3503c^g^/Rv3504	+ve
AC**TAGAAC**GT**GTTACA**TTTC	3.45E-08	Rv3515c^h^/Rv3516^i^	+ve
GT**TAGAAC**CT**GTTCTA**CTCG	3.40E-07	Rv3520c^j^/Rv3521^k^	+ve
AC**TGTAAC**GT**GTTCTA**GTTA	9.47E-09	Rv3525c/Rv3526^l^	ND
AC**TAGAAC**GT**GTTCCT**GTTT	1.79E-08	Rv3531c^m^/Rv3532	+ve
AA**TGAAAC**GT**GTTCTA**GCCT	1.42E-07	Rv3545c^n^/Rv3546°	+ve
AC**TAGAAC**AC**GTTCC****G**ATTT	1.99E-08	Rv3570c^p^/Rv3571^q^	+ve
TC**TGTAAC**AT**GTTCTA**GTTA	1.99E-08	Rv3570c^p^/Rv3571^q^	+ve
AC**TAGAAC**GT**GTTCTA**ATAG	2.35E-11	Rv3573c^r^/Rv3574^s^	+ve

a–s Orthologous genes, e.g. *MSMEG_0309*, is an orthologue of *Rv0223c.*

ND means not determined whereas –ve means no binding was observed.

### The motifs predicted are also regulated by KstR

Two approaches were used in order to obtain experimental evidence for the motif predictions in *M. smegmatis* and *M. tuberculosis*. First, RTq-PCR was used to measure the levels of expression of the flanking genes in the ΔkstR1 mutant and compare them with those in wild-type *M. smegmatis*. Second, EMSAs were used to demonstrate the binding of His_6_-KstR_Mtb_ to the predicted *M. tuberculosis* motifs.

The levels of expression from 11 of the predicted motifs in *M. smegmatis* were measured. If the predicted motif is biologically relevant, then the flanking genes should be de-repressed in the ΔkstR1 mutant. RTq-PCR analysis showed that all genes tested were significantly de-repressed in the mutant compared with the wild-type strain, with levels of de-repression ranging from 6-fold (*MSMEG_5932*) to 155-fold (*MSMEG_6038*) (Fig. 2B). EMSAs were carried out to look for binding of His_6_-KstR_MTB_ to 13 of the predicted *M. tuberculosis* motifs, and binding was observed in 12 of these (Table 3).

### Microarray analysis indicates that a large number of genes are de-repressed in the ΔkstR1 mutant

In order to obtain a genome-wide picture of genes controlled by *kstR*, we carried out competitive hybridizations between cDNA from wild-type *M. smegmatis* and the mutant strain ΔkstR1 using *M. smegmatis* microarrays. The full results of the microarray analysis are given in Table S1. Using a *P*-value of 0.05 corrected for multiple testing, a total of 132 genes were significantly upregulated (6- to 1771-fold), and 27 were downregulated (6- to 18-fold).

The microarray analysis showed de-repression of genes flanking 26 of the 31 motifs that we had identified in *M. smegmatis* (Table 4). For the other five, although the computational analysis indicates the presence of a motif, a combination of low levels of de-repression, low levels of significance in terms of gene expression changes, and the absence of an orthologous gene with a motif in *M. tuberculosis* suggests that these instances of the motif may not be biologically relevant.

**Table 4:**
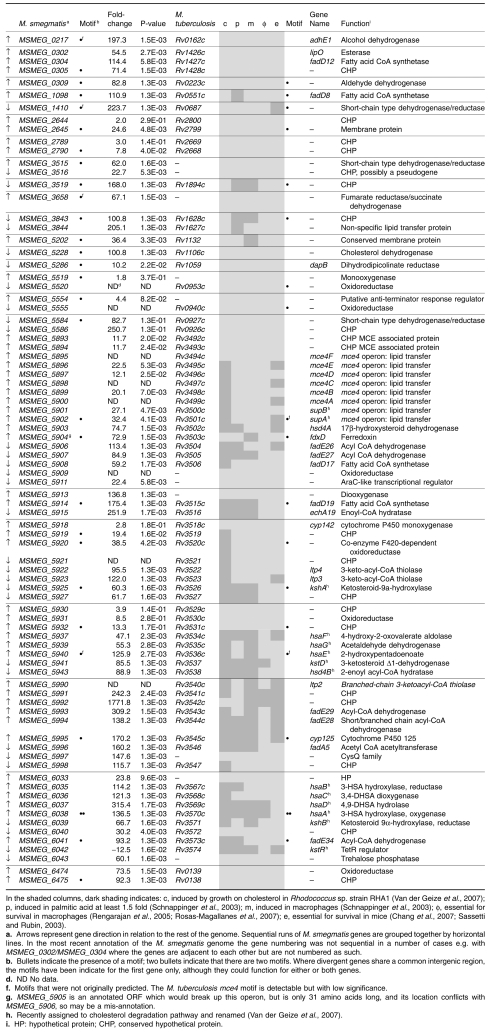
The *kstR* regulon.

We identified four additional instances of the motif (*MSMEG_0217, MSMEG_1410*, *MSMEG_3658* and *MSMEG_5940*) that had not been picked up in the original search (Table 4). Three of these were found to overlap with coding sequences of adjacent genes and one (*MSMEG_1410*) was within an operon, and therefore would have been excluded from our original search. We also searched *M. tuberculosis* in regions where motifs were present in *M. smegmatis*, and found possible matches for two of these (*Rv3501c* and *Rv3536c*) but with a relatively low probability as determined by MAST.

### Defining the *kstR* regulon in *M. smegmatis*

The genes with altered expression in the microarray analysis will be a combination of those where binding of KstR directly affects transcription (the *kstR* regulon), and those that are secondary effects. The genes in the *kstR*_Msm_ regulon were defined by using a combination of data from the motif search (Table 3), EMSA analyses (Table 3), RTq-PCR analyses (Fig. 2A and B) and genome-wide expression data from the microarray studies (looking both at fold-change and *P*-value; Table S1), comparative genomics with *M. tuberculosis*, and examination of operon organisation. Most of the genes we have included are clearly supported by most or all factors. We have in a minority of cases included genes where there are no microarray data, or where the array data are not significant at the 0.05 level, but the fold-change and other factors support their inclusion. The *kstR*_Msm_ regulon, containing 83 genes, is listed in Table 4.

### Transcriptional changes not associated with a KstR motif

We also analysed the genes where expression changes were not associated with a KstR motif, which are likely to be secondary effects. We examined fold-change, *P*-value and genomic organization, and took runs of modulated genes into account and included 99 genes in this group. Most of these (87) were upregulated in the mutant (2- to 100-fold) and 11 were downregulated (5- to 16-fold) (Table S1); 74 of them (all but two induced in the mutant) lie in putative operons. The co-regulation of adjacent genes is particularly strong evidence that the effect is a genuine indirect effect of the *kstR* deletion, rather than being due to the noise inherent in microarray experiments.

### Predicting the kstR regulon in *M. tuberculosis*

There are clear orthologues of most of the genes in the *kstR*_Msm_ regulon in *M. tuberculosis* (Table 4), and these include all the genes in *M. tuberculosis* that were predicted to lie downstream of a motif (Table 3). The presence of the motif and the orthology with the de-repressed *M. smegmatis* gene is robust evidence for inclusion of these genes in the *M. tuberculosis kstR* regulon. The most striking observation from the data is that there is a large region in both mycobacterial genomes [*Rv3492c* to *Rv3574* (*kstR*) and *MSMEG_5893* to *MSMEG_6043*] that contains a number of operons that are de-repressed and associated with a motif.

### Functional analysis of the *kstR* regulon

Analysis of the functions of the genes in the *kstR* regulon was carried out by a combination of blast analyses, as well as searching the Tuberculist database (http://genolist.pasteur.fr/TubercuList/) and the literature. It was striking that the predicted functions of most of the genesin the regulon relate to lipid metabolism or to redox reactions. For example, there are 10 *fad* genes (*fadA5*, *fadE26*, *fadE27*, *fadE29*, *fadE28*, *fadE34*, *fadD8*, *fadD12*, *fadD17* and *fadD19*), one *ech* gene (echA19), one *lip* gene (*lipO*), and at least three keto acyl-CoA thiolases (*ltp2*, *ltp3* and *ltp4*). In addition, the *mce4* operon is part of this regulon, and it has been suggested that the *mce* operons are involved in the import of lipids or are lipid-associated (Mitra *et al*., 2005; Rosas-Magallanes *et al*., 2007; Van der Geize *et al*., 2007). Other genes in the regulon, *hsaC* and *hsaD* (formerly *bphC* and *bphD* respectively), have been implicated in both cell wall synthesis (Anderton *et al*., 2006) and cholesterol degradation (Van der Geize *et al*., 2007).

The *kstR* regulon, particularly the region *Rv3492c* to *Rv3574 (kstR*) (Table 4), contains a large number of genes that have been found by others to be induced in macrophages (Schnappinger *et al*., 2003), essential for survival in both macrophages and mice by genome-wide essentiality studies (Sassetti and Rubin, 2003; Rengarajan *et al*., 2005) and induced by growth on lipids, such as palmitic acid and cholesterol (Schnappinger *et al*., 2003; Van der Geize *et al*., 2007) (Table 4). The relevance of this observation and of the role of the *kstR* regulon *in vivo* is discussed below.

### Functional analysis of KstR-independent genes

Of the 99 genes that showed transcriptional changes but were not associated with a motif, some were also predicted to be involved in lipid metabolism. Of the others, 31 are predicted to be involved in translation, while other groups included chaperones, the pentose phosphate pathway, dipeptide transport and glycerol metabolism. In addition, a group of genes (*MSMEG_0065* to *MSMEG_0068*) show homology to the *M. tuberculosis esxAB* gene cluster, which is important for virulence.

## Discussion

Lipid metabolism (both anabolic and catabolic) plays a key role in the pathogenesis of *M. tuberculosis*. Mycobacteria and other prokaryotes are able to use fatty acids as a sole carbon source via β-oxidation, and these pathways are thought to be particularly important for the survival of *M. tuberculosis in vivo* (Bishai, 2000; McKinney *et al*., 2000). In addition to using fatty acids as a carbon source during infection, cell wall lipids play a variety of roles in pathogenesis (Russell *et al*., 2002; Rao *et al*., 2006).

We have shown that *kstR*, a transcriptional regulator highly conserved within the actinomycetes, controls the expression of a number of genes involved in lipid metabolism in both *M. tuberculosis* and *M. smegmatis*. We have identified a conserved 14 bp palindromic motif in the promoter regions of genes in the regulon and demonstrated binding of purified KstR to 29 bp DNA probes containing the motif. We have not formally demonstrated binding of KstR to the motif itself (as opposed to the 7–8 bp flanking sequences), but the correlation of the microarray data with the motif location, the demonstration that KstR binds to several 29 bp probes that have the motif as the only common feature, and the fact that other TetR regulators have been shown to bind to short palindromic sequences is compelling evidence that it is the motif that is bound by KstR, and not the flanking sequences. Although we cannot conclude how repression occurs in all cases without mapping the transcription start sites, there are instances where the motif overlaps or lies inside coding regions (*MSMEG_0217 MSMEG_1410*, *MSMEG_3658* and *MSMEG_5940*), suggesting that KstR physically blocks RNA polymerase progression. We have deduced a hypothetical *M. tuberculosis kstR* regulon using the *M. smegmatis* microarray data. The high degree of syntenic similarity, the conservation of KstR motifs, and the demonstration from other groups that the regulon is induced under relevant conditions (Schnappinger *et al*., 2003) give us confidence that the majority of this regulon is correct.

A large number of genes controlled by *kstR* are found within a cluster of genes adjacent to the *kstR* gene (Table 4). This region was also the focus of a recent study which identified the genes induced by growth of *Rhodococcus* on cholesterol (Van der Geize *et al*., 2007). The authors assigned 28 genes to the cholesterol degradation pathway, mostly through bioinformatics, but also with experimental verification of some candidate genes. Although they named *Rv3574 kstR* (for ketosteroid regulator), no experimental evidence was provided as to the role of this gene. Our results show that most of these genes are indeed controlled by *kstR* and have the *kstR* motif in their promoter regions. We have confirmed that the binding motif identified here is also present in appropriate sites in the *Rhodococcus* genome (data not shown).

Despite the proposed involvement of the rhodococcal *kstR* regulon in cholesterol degradation, we suggest that the situation is more complex in mycobacteria. First, we have demonstrated that the regulon is extremely large (83 genes in *M. smegmatis*), and it is unlikely that so many genes are required just for cholesterol utilization. Second, palmitic acid also induces 22 genes (including *kstR* itself) in the *kstR* regulon in *M. tuberculosis* (Table 4). We propose therefore that the *kstR* regulon is involved in the uptake and utilization of a variety of lipids, of which cholesterol is just one. It can be argued that it makes biological sense for the bacteria to have a mechanism that will enable degradation of a variety of lipids. We have elected to retain the name *kstR* because it is relevant to at least part of the function, and in order to reduce confusion. In *Rhodoccocus*, all 51 genes in the region orthologous to *Rv3492c–Rv3574* were upregulated in the presence of cholesterol, whereas we found that not all of these were induced in the *M. smegmatis kstR* mutant (Table 4). This suggests that part of the cholesterol response is under the control of regulators other than *kstR*. The induction ratios seen in the presence of cholesterol and palmitate are lower than we observed in this study, and this may reflect low intracellular concentrations of the molecules, or that they de-repress the regulon with different affinities.

It is noteworthy that 18 of the genes in the *kstR* regulon have been shown to be essential *in vivo* in mouse or macrophage models (Table 4). These include *kstR* itself, and some of the *mce4* operon genes. Many of these essentiality studies used the genome-wide TraSH screen, where methodological and statistical noise causes some error. However, the TraSH methodology has been shown to be reasonably robust through validation of individual genes. There is already good evidence that the *mce4* operon is required *in vivo* for survival in mice (Joshi *et al*., 2006) and macrophages (Rosas-Magallanes *et al*., 2007). In addition, deletion of the *Rv3540c–Rv3545c* operon causes attenuation of growth in macrophages and immunocompetent mice (Chang *et al*., 2007). Presumably the reason for the essentiality for *kstR* (where a mutant will express all the normally regulated genes constitutively) differs from the other genes (where a mutation results in loss of function). The induction levels we saw in the *kstR* regulon were extremely high, so the essentiality of *kstR* may merely reflect the energy cost of the elevated gene expression; alternatively, there may be times in the infection process where expression of a particular gene in the regulon is detrimental for another reason.

We identified 99 genes that were induced or repressed in the mutant but did not appear to be directly regulated by *kstR* (Table S4). Some of these are involved in lipid metabolism, suggesting the involvement of other regulatory systems, but many were ribosomal and chaperone genes. The induction of ribosomal and chaperone genes suggests that the transcription levels achieved by knocking out *kstR* place a strain on the translation apparatus of the cell. This may explain the slight growth defect seen in the mutant. It is possible that this situation does not occur in reality, and the transcription levels achieved by de-repression in the presence of an inducer will not be as high, so that less stress will be put on the translational apparatus.

The *mce4* operon appears to be a key part of the *kstR* regulon in *M. smegmatis*. Circumstantial data are accumulating that the *mce* operons (of which *M. tuberculosis* has four, and *M. smegmatis* at least five) function as lipid transport systems (Santangelo *et al*., 2002; Mitra *et al*., 2005; Uchida *et al*., 2007; Van der Geize *et al*., 2007). The results presented here show that the *mce4* operon is co-regulated with other genes involved in fatty acid metabolism, and support the hypothesis that the *mce* genes are involved in lipid uptake. Apart from its use as an energy source, cholesterol has been implicated in the uptake of *M. tuberculosis* by macrophages (Gatfield and Pieters, 2000), although the receptor for host cholesterol is unknown. It is tempting to suggest that the *mce4* system might play a role in the bacterial–host interaction, if it is also involved in internalizing cholesterol (Arruda *et al*., 1993; Casali *et al*., 2002; Mitra *et al*., 2005).

KstR is a TetR-type regulator; in this paradigm, repression is controlled by the binding of an inducer molecule. TetR itself binds tetracycline (Ramos *et al*., 2005), and ligands for other repressors are often hydrophobic molecules (Frenois *et al*., 2004). The induction of the *kstR* regulon by palmitate and cholesterol supports the hypothesis for a fatty acid ligand. Additionally, the induction of the regulon upon entry into the macrophage, and the essentiality of many of the genes in the regulon for *in vivo* survival (Table 4), suggests that the ligand(s) are present inside the host.

While it is likely that the *kstR* regulon has a major catabolic role, it is possible that some of the genes in the regulon are anabolic, although we did not see differences in quantity and abundance of the major cell wall lipids (data not shown). One gene that is present in the *kstR* regulon of *M. tuberculosis* but not that of *M. smegmatis*, is the *nat* gene encoding arylamine N-acetyltransferase (Anderton *et al*., 2006). Mutants lacking *nat* are defective in mycolic acid synthesis (Bhakta *et al*., 2004), indicating a possible anabolic role for some genes in the *kstR* regulon. The Nat protein can bind to the antitubercular drug isoniazid, reducing its efficacy (Sandy *et al*., 2002). The induction of the *kstR* regulon in *M. tuberculosis in vivo* may therefore partially affect the antibiotic resistance of the bacteria.

In conclusion, we have described a large regulon within the mycobacteria. In *M. tuberculosis*, this makes up almost 2% of the genome. Although at least the core of this regulon is highly conserved in non-pathogens, many of the genes are critical in the pathogenesis of *M. tuberculosis*. Investigating both the regulation of *kstR* and the functions of the genes in the regulon is likely to provide important new information in our understanding of the adaptation of this major pathogen to its host.

## Experimental procedures

### Bacterial strains and culture conditions

Cultures of *M. smegmatis* mc^2^155 were grown at 37°C with shaking in Middlebrook 7H9 broth (Difco) containing 10% oleic acid-albumin-dextrose-catalase supplement (Becton Dickinson) and 0.05% Tween 80. Hygromycin (50 μg μl^−1^), kanamycin (20 μg μl^−1^), 5-bromo-4-chloro-3-indolyl-β-D-galactopyranoside (Xgal, 50 μg μl^−1^) and sucrose (2% w/v) were used for selection as appropriate. *E. coli* DH5α was used as a host for cloning, and *E. coli* BL21(DE3) (Novagen) was used as a host for expression of recombinant KstR_Mtb_. Both *E. coli* strains were grown in Luria–Bertani, and kanamycin (50 μg μl^−1^) was used for plasmid selection and maintenance. The strains and plasmids used in this study are described in Table 1.

**Table 1:** Bacterial strains and plasmids used in this study.

Strain/plasmid	Genotype/description	Source
Strain		
*E. coli*		
DH5α	*supE44*Δ*lacU169* (*φlacZΔM15*) *hsdR17 recA1*	Invitrogen
*BL21*(DE3)	*OmpT hsdS*_*B*_*(r*_*B*_^–^*m*_*B*_^–^*) gal dcm* (DE3)	Novagen
*M. smegmatis*		
mc^2^155	High-frequency transformation mutant ATCC 607	Snapper *et al*. (1990)
ΔkstR1	Δ*kstR*_Msm_	This study
Plasmid		
p2NIL	Gene manipulation vector, *Kan*	Parish and Stoker (2000)
pGOAL19	*PacI* cassette vector, *hyg* P_ag85_-*lacZ* P_hsp60_-*sacB*,	Parish and Stoker (2000)
pET30a	*E. coli* expression vector, *Kan*	Novagen
pCS1	3.5 kb fragment containing *kstR*_Msm_ in p2NIL	This study
pCS2	646 bp deletion of pCS1	This study
pCS3	pCS2 with the pGOAL19 *PacI* cassette inserted	This study
pSK35	*kstR*_Mtb_ in pET30a expression vector	This study

### Deletion of *kstR*_*Msm*_ by homologous recombination

A 646 bp deletion in *MSMEG_6042*(*kstR*_Msm_) was made in *M. smegmatis* mc^2^155 by homologous recombination (Parish and Stoker, 2000). Briefly, a 3.5 kb fragment containing the entire *kstR*_*Msm*_ gene and flanking regions was PCR amplified from mc^2^155 genomic DNA using Δ*kstR*_Msm_ forward and reverse primers (Table 2). The primers had BamHI–HindIII sites (shown in upper case in Table 2) introduced into them in order to enable cloning of the 3.5 kb fragment into p2NIL, resulting in plasmid pCS1. A deletion was made in pCS1 by inverse PCR using inv_*kstR*_Msm_ forward and reverse primers (Table 2) and religation of the BglII-digested PCR fragment. One of the BglII sites was present in the genome, and the other was introduced in the inv_*kstR*_Msm_ reverse primer. During the writing of this manuscript, the *M. smegmatis* genome was re-annotated and *kstR*_Msm_ was designated as being 66 bp shorter than annotated previously. These primers were designed to remove 646 bp from the coding sequence. The deletion removes 39 bp upstream of the coding sequence according to the new annotation. Neither annotation has been confirmed experimentally. The deletion in the resulting plasmid pCS2 was confirmed by sequencing (sequencing reactions performed by MWG Biotech) across the junction (data not shown). Finally, the PacI cassette was inserted into pCS2, resulting in the suicide delivery vector pCS3.

pCS3 was electroporated into competent mc^2^155 (Parish and Stoker, 1998), and single cross-overs were selected for on medium containing hygromycin, kanamycin and Xgal. A single blue kanamycin and hygromycin-resistant colony was streaked onto fresh media without any selective markers, and incubated at 37°C for 3–5 days to allow the second cross-over to occur. Serial dilutions were plated onto media containing sucrose and Xgal to select for double cross-overs. Potential double cross-overs (white sucrose-resistant colonies) were screened for kanamycin sensitivity and confirmed by colony PCR. The resulting mutant was called ΔkstR1. The intergenic region between *fadE34* and *kstR* was sequenced in order to confirm that the promoter had not been affected by the mutagenesis.

### RNA extraction

RNA for microarray analysis and RTq-PCR was extracted from both wild-type mc^2^155 and ΔkstR1 strains by direct sampling into guanidinium thiocyanate (GTC). Briefly, 10 ml of aerated cultures in logarithmic phase (OD_600_ 0.4–0.5) was added to 40 ml of 5 M GTC to prevent further transcription. The culture was pelleted by centrifugation (20 min, 4000 *g*, 4°C) and resuspended in 200 μl of water. The cultures were transferred to screwcap tubes containing 0.5 ml of 0.1 mm zirconia/silica beads (Biospec), and 700 μl of buffer RLT (Qiagen) was added. The bacteria were lysed using a Mini-BeadBeater^TM^ (BioSpec), and cell lysates were recovered by centrifugation (5 min, 13 000 *g*, 4°C). RNA was purified from the lysate using an RNeasy kit (Qiagen) and treated with DNase (Qiagen) according to the manufacturer's instructions. Finally, the samples were eluted in 30 μl of RNase-free water, and quantity was assessed using a NanoDrop (NanoDrop technologies).

### Reverse transcription reactions for RTq-PCR

Real-time quantitative polymerase chain reaction was used for the analysis of the expression of single genes. Prior to reverse transcription, RNA was treated with DNase (Invitrogen) for 30 min at 37°C, followed by heat inactivation. Reverse transcription took place in a total volume of 20 μl containing 100 ng total RNA, 300 ng random primers (Invitrogen), 10 mM DTT, 0.5 mM each of dCTP, dATP, dGTP and dTTP, and 200 units of Superscript III (Invitrogen). For primer annealing, RNA and random primers were heated to 65°C for 10 min in a volume of 13 μl and then snap-cooled on ice prior to the addition of the remaining components. For reverse transcription, the reactions were incubated at 55°C for 50 min. A total of 1 μl (equivalent to 5 ng of RNA) of cDNA was used in the RTq-PCRs.

### Real-time quantitative polymerase chain reaction

Real-time quantitative polymerase chain reactions were set up using the DyNAmo SYBR Green qPCR kit (MJ Research), and RTq-PCR was performed using the DNA Engine Opticon® 2 System (GRI). 20 μl reactions were set up on ice containing 1× DNA Master SYBR Green I mix, 1 μl of cDNA product and 0.3 μM of each primer. Sequences of each primer are given in Table 2. Reactions were heated to 95°C for 10 min before cycling for 35 cycles of 95°C for 30 s, 62°C for 20 s, and 72°C for 20 s. Fluorescence was captured at the end of each cycle after heating to 80°C to ensure the denaturation of primer-dimers. At the end of the PCR, melting curve analysis was performed and PCR products were analysed on an agarose gel to ensure product specificity. The experiment was performed in triplicate and each gene was measured in duplicate, giving a total of six data points per gene.

### Expression and purification of recombinant KstR_Mtb_

The *kstR*_Mtb_ gene was PCR amplified from *M. tuberculosis* H37Rv genomic DNA using pET_*kstR*_Mtb_ forward and reverse primers (Table 2). These primers had NcoI–HindIII sites introduced into them to allow for cloning into the pET30a expression vector. The nucleotide sequences corresponding to the restriction sites are shown in upper case in Table 2, and the start site of *kstR*_Mtb_ is underlined (in accordance with the old annotation). The resulting plasmid, pSK35, was sequence verified and used for expression and purification of C-terminally His-tagged KstR_Mtb_. For expression, *E. coli* BL21(DE3) cultures containing plasmid pSK35 were grown at 37°C until mid-logarithmic phase. Cultures were induced with 1 mM IPTG (isopropyl-beta-D-thiogalactopyranoside) for 2 h at 37°C and harvested by centrifugation (10 min, 4000 *g*, 4°C). The cell pellet was resuspended in 5 ml of lysis buffer (20 mM HEPES pH 8.0, 150 mM NaCl, 1 mM β-mercaptoethanol, 10 mM imidazole) and lysed by passage through a cell disrupter (Constant Systems) set at 18 kpsi. The lysate was centrifuged (25 min, 16 000 *g*, 4°C) and His_6_-KstR_Mtb_ from the soluble fraction was purified by immobilized metal ion affinity chromatography using a HiTrap Ni-NTA column (GE Healthcare Biosciences), followed by SEC using a Superdex200 10/30 column (GE Healthcare Biosciences).

### Electrophoretic mobility shift assays

Oligonucleotides (Table 2) were annealed by heating to 95°C for 10 min and allowed to cool slowly to room temperature. The resulting probes were end-labelled with DIG-11-ddUTP using the DIG gel shift kit, 2nd generation (Roche), according to the manufacturer's instructions. For the binding reaction, varying amounts of purified His_6_-KstR_Mtb_ were incubated with 0.66 pmol of labelled fragment in binding buffer [20 mM HEPES pH 8.0, 75 mM NaCl, 10 mM MgCl_2,_ 0.1 μg of poly-l-lysine, 1 μg of poly(dI-dC)]. Specific and non-specific competitors were added for the control reactions. Specific competition reaction mixtures contained a 100-fold excess of unlabelled probe, and non-specific competition reaction mixtures contained a 150-fold excess of poly(dI-dC). Incubations were carried out for 30 min at room temperature, and reaction mixtures were loaded onto 8% polyacrylamide gels containing 0.5× TBE. Gels were run, with cooling at 80–100 V over 1.5–2 h. The DNA–protein complexes were contact blotted onto positively charged Hybond-N+ nylon membranes (Amersham), and detected by anti-DIG-alkaline phosphatase and the chemiluminescent substrate CSPD as described by the manufacturer (Roche). Membranes were exposed to X-ray film at room temperature for 10–30 min.

### Molecular weight determination of the protein–DNA complex by SEC

The molecular weight of His_6_-KstR_Mtb_ was determined by analytical SEC on a Superdex200 10/30 column. A standard curve of *v*_*e*_*/v*_*o*_ was constructed using the peak elution volume (*v*_*e*_) of the following standards: ovalbumin (43.0 kDa), ribonuclease A (13.7 kDa), albumin (67.0 kDa), chymotrypsinogen A (25.0 kDa) and catalase (232.0 kDa). The void volume (*v*_*o*_) of the column was determined with blue Dextran 2000. All SEC experiments were performed at a flow rate of 0.5 ml min^−1^ in 20 mM HEPES pH 8.0, 75 mM NaCl, 10 mM MgCl_2_ and 1 mM β-mercaptoethanol. His_6_-KstR_Mtb_ was used at a concentration of 15 μM. Samples containing His_6_-KstR_Mtb_ and the 29 bp annealed probes (Table 2) were incubated on ice for 15 min prior to analysis. Collected fractions were analysed by SDS-PAGE and stained with Coomassie blue and ethidium bromide to confirm the presence of protein and DNA.

### Microarray analysis of *M. smegmatis*ΔkstR1

Microarrays for genome-wide expression analysis of the mutant strain ΔkstR1 were obtained from the Pathogen Functional Genomics Resource Centre at TIGR (http://pfgrc.tigr.org/). The arrays consist of 6746 different 70-mer single-stranded oligonucleotides spotted onto glass slides. The oligonucleotides represent the entire *M. smegmatis* genome, and each oligonucleotide is spotted four times. Wild-type RNA was competitively hybridised against mutant RNA, and the design included a dye-swap. For the labelling reactions, 2–10 μg of RNA was labelled with either Cy3-dCTP or Cy5-dCTP (Amersham Pharmacia Biotech). In each case, 3 μg of random primers (Invitrogen™ Life Technologies) was annealed to the RNA by heating to 95°C for 5 min, followed by snap-cooling on ice. The labelling reaction contained 0.5 mM each of dATP, dGTP and dTTP, 0.2 mM dCTP, 10 mM DTT, 60 μmol of Cy3-dCTP (or Cy5-dCTP) and 500 units of Superscript II (Invitrogen™ Life Technologies) in a final volume of 25 μl. The samples were incubated in for 10 min at 25°C, followed by a 90 min incubation at 42°C in the dark.

The slides were prehybridised by incubating in prehybridisation buffer (3.5× SSC, 0.1% SDS, 10 mg ml^−1^ BSA) at 65°C for 20 min. They were then washed in 400 ml of water, followed by 400 ml of isopropanol, for 1 min each. The slides were dried by centrifugation (1500 *g*, 5 min, room temperature) and stored in the dark until hybridization (< 1 h).

### Microarray hybridisations

Labelled wild-type samples were combined with the corresponding labelled mutant samples, and were purified using a MinElute PCR Purification Kit from Qiagen. Samples were eluted in 25 μl of water and hybridised onto the array in hybridisation buffer (4× SSC, 40% formamide, 0.1% SDS). The samples were denatured by heating to 95°C for 2 min before being added to the array. Hybridization took place under a glass coverslips in a humidified slide chamber (Corning) submerged in a 65°C water bath for approximately 16 h. Coverslips were removed in wash buffer I (1× SSC, 0.05% SDS) prewarmed to 65°C, and slides were washed sequentially in buffer I at 65°C for 2 min, followed by two washes in buffer II (0.06× SSC) at room temperature for 2 min each. Slides were dried by centrifugation (1500 *g*, 5 min, room temperature), and were scanned using an Affymetrix 4I8 scanner. The image files were quantified using ImaGene 7.0 software (BioDiscovery). The whole experiment was performed in duplicate, and two arrays were used per experiment. As the oligonucleotides were spotted four times on the slides, this gave us a total of eight data points per open reading frame (ORF).

### Microarray data analysis

Data analysis was performed using functions from the limma (linear models for microarray data analysis) (Smyth, 2005) (http://bioinf.wehi.edu.au/limma/) and yasma (Wernisch *et al*., 2003) software packages. Differentially expressed genes were identified by linear models using an experimental design for two-colour arrays which incorporated biological replicates with dye-swapped technical replicates. Data for control spots, and for spots with expression levels in the lower 10% quantile, were discarded. This was followed by background correction and rank normalization. Duplicate spots within the arrays were averaged before performing the linear model fit. False discovery rate adjustment was made using Benjamini and Hochberg's method (1995), and genes were considered significant if they had an adjusted *P*-value less than 0.05.

### Bioinformatic analyses

The identification of orthologues of *kstR*_Mtb_, and other comparative genomic and operon organization analyses, were carried out using ACT (Carver *et al*., 2005). Sequence alignments were performed using ClustalW (Thompson *et al*., 1994). Motif analysis was carried out using MEME (Bailey and Elkan, 1994) and MAST (Bailey and Gribskov, 1998). Weblogo version 3 beta (Crooks *et al*., 2004) (http://weblogo.berkeley.edu/) was used to derive the image in Fig. 3B.

### Lipid extraction and analyses

Polar and apolar lipids were extracted from *M. smegmatis* strains according to established procedures (Burguiere *et al*., 2005), and were analysed using thin-layer chromatography as detailed previously (Dobson *et al*., 1985). The cell wall-bound mycolic acids from the above delipidated extracts were analysed as described previously (Alderwick *et al*., 2005).

## Supplementary material

The following supplementary material is available for this article:

Table S1-S4 **Table S1.** All genes. Normalised microarray data for all M. smegmatis genes for which valid data were obtained (kstR mutant compared to the wild-type strain). **Table S2.** Genes, P<0.05. Normalised microarray data for all M. smegmaits genes found to be significantly altered in expression in the kstR mutant compared to the wild-type strain. **Table S3.** kstR regulon. Normalised microarray data for M. smegmatis genes we predict to be directly controlled by kstR (kstR mutant compared to the wild-type strain). **Table S4.** Secondary effects. Normalised microarray data for M. smegmatis genes were significantly altered in expression (kstR mutant compared to the wild-type strain), but which we believe not to be directly controlled by kstR.

This material is available as part of the online article from: http://www.blackwell-synergy.com/doi/abs/10.1111/j.1365-2958.2007.05827.x
